# 1-Ethyl-4-isopropyl-1,2,4-triazolium bromide

**DOI:** 10.1107/S2414314623007848

**Published:** 2023-09-14

**Authors:** Aaron Maynard, Taylor M. Keller, Michael Gau, Daniel R. Albert, Edward Rajaseelan

**Affiliations:** a Department of Chemistry, Millersville University, Millersville, PA 17551, USA; b Department of Chemistry, University of Pennsylvania, Philadelphia, PA 19104, USA; University of Aberdeen, United Kingdom

**Keywords:** crystal structure, triazolium salt, heterocyclic ionic compound

## Abstract

An ionic compound consisting of a triazolium cation and bromide anion, C_7_H_14_N_3_^+^·Br^−^, has been synthesized and structurally characterized using single-crystal X-ray diffraction and NMR. The compound crystallizes in the monoclinic space group *P*2_1_/*m* with the non-hydrogen atoms of one cation lying on general positions and the others lying on a mirror plane. One bromide ion also lies on the mirror. The extended structure exhibits only weak inter­molecular inter­actions between heterocyclic C—H groups and Br^−^ ions.

## Abstract 2

The title compound crystallizes with *Z* = 6 in space group *P*2_1_/*m*.

## Graphical abstract

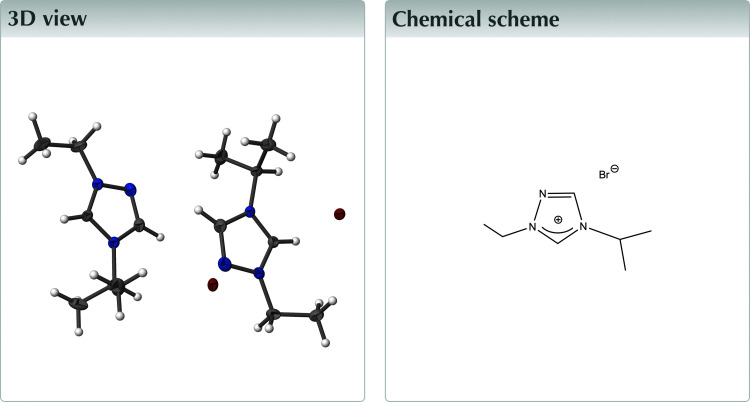

## Structure description

Asymmetric 1,2,4-triazolium cations are of inter­est due to their utility as cations in ionic liquids (ILs) and as precursors to N-heterocyclic carbenes (NHCs) (Dwivedi *et al.*, 2014 ▸; Nelson, 2015 ▸; Strassner *et al.*, 2013 ▸; Riederer *et al.*, 2011 ▸; Chianese *et al.*, 2004 ▸). The crystal structures of several triazolium salts have been reported (Peña Hueso *et al.*, 2022 ▸; Kumasaki *et al.*, 2021 ▸; Ponjan *et al.*, 2020 ▸; Guino-o *et al.*, 2015 ▸). We have synthesized many imidazolium and triazolium salts as precursors in the synthesis of NHC complexes of rhodium and iridium (Castaldi *et al.*, 2021 ▸; Gnanamgari *et al.*, 2007 ▸; Idrees *et al.*, 2017 ▸; Nichol *et al.*, 2011 ▸; Newman *et al.*, 2021 ▸; Rushlow *et al.*, 2022 ▸).

**Figure 1:**
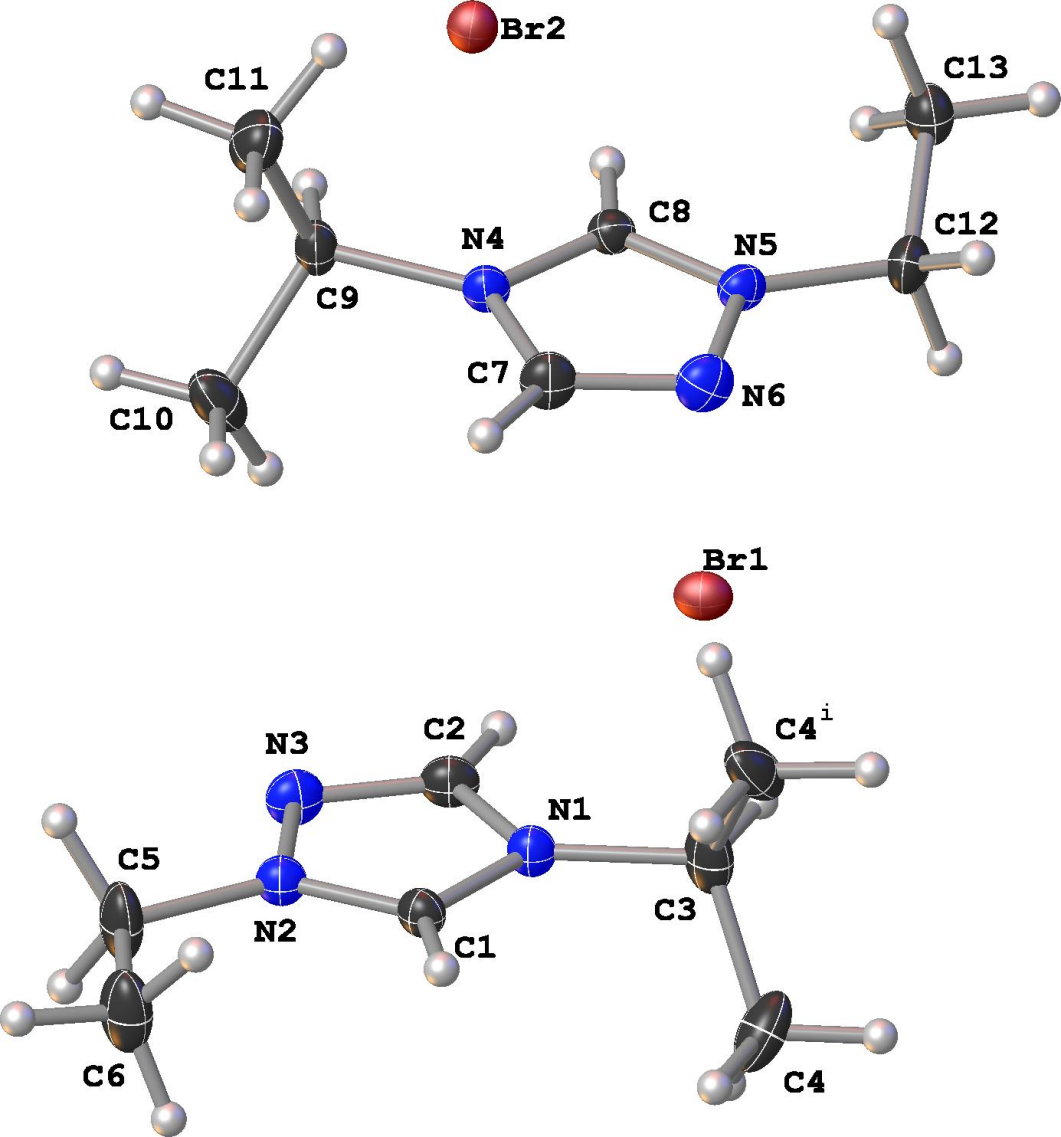
The mol­ecular structure of the title compound with displacement ellipsoids drawn at the 50% probability level. The N1 mol­ecule (except C4) and Br1 lie on the (*x*, 3/4, *z*) mirror plane. Atom C4^i^ is generated by the symmetry operation *x*, 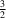 − *y*, *z*.

The mol­ecular structure of the title compound is shown in Fig. 1 ▸. There are one and a half mol­ecules in the asymmetric unit with the non-hydrogen atoms of the N1 cation (except C4) and Br1 lying on the (*x*, 3/4, *z*) mirror plane. All the atoms of the N4 cation and Br2 occupy general positions. The bond lengths in the triazolium rings indicate aromaticity with C—N bonds exhibiting distances in the range of 1.305 (2)–1.366 (2) Å and N—N bond distances near 1.365 Å; the N—C—N bond angles in the triazolium ring range from 106.93 (18) to 111.35 (18)°. The C1—N2—C5—C6 torsion angle of the ethyl side chain in the N1 cation is constrained to be 0° by symmetry and the corresponding C8—N5—C12—C13 torsion angle in the N4 cation is 24.4 (2)°.

**Figure 2:**
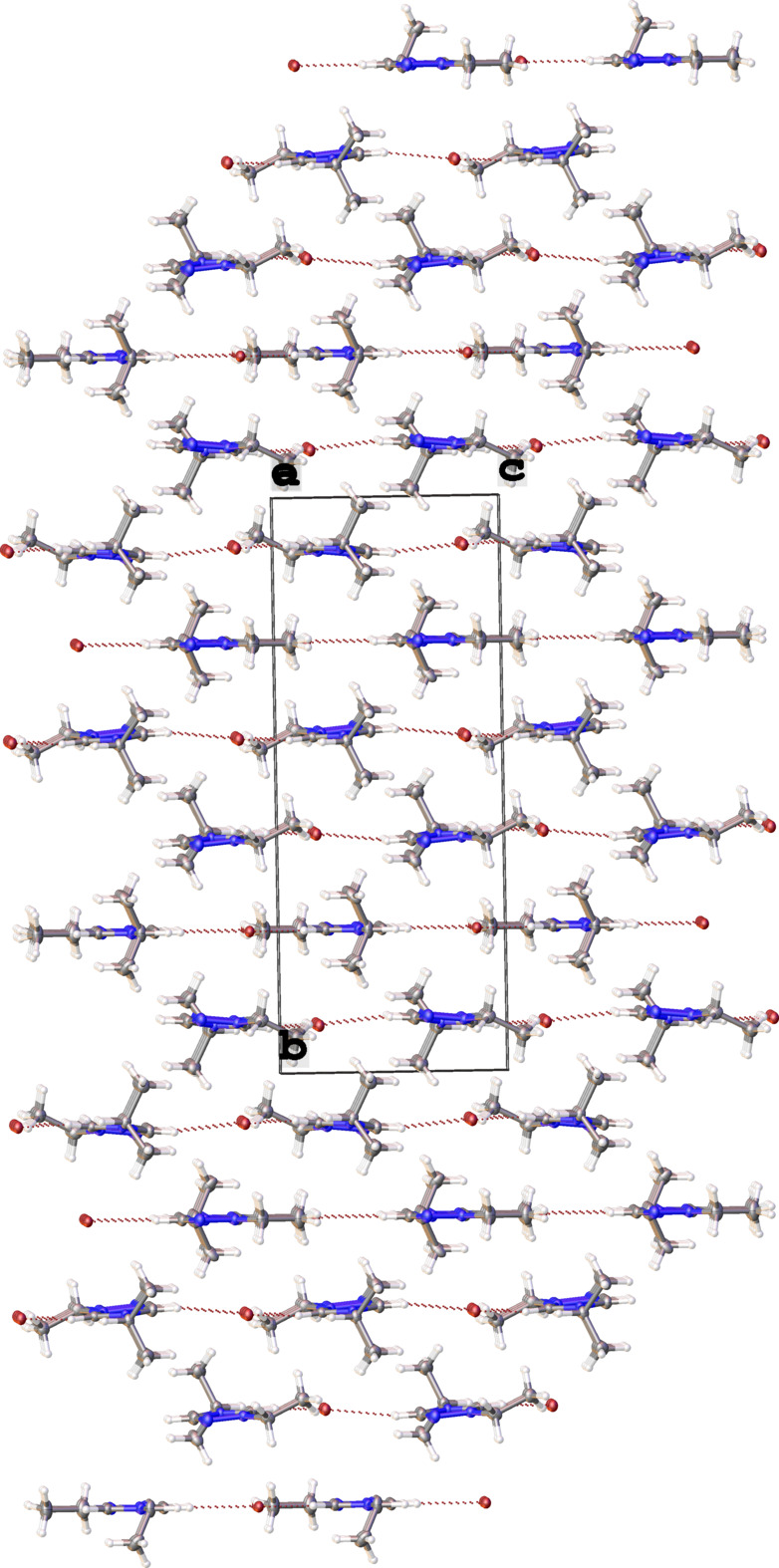
Crystal packing of the title compound shown along the *a* axis. Non-classical C—H⋯Br hydrogen-bonding inter­actions are shown as dotted red lines.

The crystal packing of the title compound is displayed in Fig. 2 ▸. There are weak non-classical hydrogen-bonding inter­actions between the heterocyclic C—H groupings and bromide ions. These weak inter­actions are shown as dotted red lines in Fig. 2 ▸ and summarized in Table 1 ▸.

## Synthesis and crystallization

1-Ethyl triazole was purchased from AmBeed. All other compounds used in the syntheses of the title compound were obtained from Sigma-Aldrich. All materials in the synthesis were used as received. The synthesis was performed under nitro­gen using reagent grade solvents, which were used as received without further purification. NMR spectra were recorded at room temperature in CDCl_3_ on a 400 MHz Varian spectrometer and referenced to the residual solvent peak (δ in p.p.m.).

1-Ethyl-1,2,4-triazole (2.01 g, 20.61 mmol) and isopropyl bromide (10.14 g, 82.4 mmol) were added to toluene (20 ml) and the mixture was refluxed for 48 h. Once cooled, the liquid was deca­nted, the white solid product that formed was washed with ether, filtered, and dried. The title compound crystallized as clear needles by slow diffusion of pentane into a CH_2_Cl_2_ solution. Yield: 1.04 g (23%). ^1^H NMR: CDCl_3_, δ (p.p.m.) 11.99 (*s*, 1 H, N—C_5_H—N), 8.85 (*s*, 1 H, N—C_3_H—N), 5.13 (*m*, 1 H, CH(CH_3_)_2_), 4.63 (*q*, 2 H, N—CH_2_), 1.74 (*d*, 6 H, CH(CH_3_)_2_), 1.65 (*t*, 3 H, CH_2_CH_3_). ^13^C NMR: δ (p.p.m.) 142.27 (N—CH—N), 141.84 (N—CH—N), 53.15 [CH(CH_3_)_2_], 48.36 (N—CH_2_), 23.14 [CH(CH_3_)_2_], 14.22 (N—CH_2_CH_3_).

## Refinement

Crystal data, data collection, and structure refinement details are summarized in Table 2 ▸.

## Supplementary Material

Crystal structure: contains datablock(s) I. DOI: 10.1107/S2414314623007848/hb4448sup1.cif

Structure factors: contains datablock(s) I. DOI: 10.1107/S2414314623007848/hb4448Isup2.hkl

Supporting information file. DOI: 10.1107/S2414314623007848/hb4448Isup3.cml

CCDC reference: 2293675

Additional supporting information:  crystallographic information; 3D view; checkCIF report

## Figures and Tables

**Table 1:** Hydrogen-bond geometry (Å, °)

*D*—H⋯*A*	*D*—H	H⋯*A*	*D*⋯*A*	*D*—H⋯*A*
C1—H1⋯Br1^i^	0.95	2.67	3.610 (2)	170
C2—H2⋯Br2^i^	0.95	2.70	3.6344 (18)	166
C7—H7⋯Br2^i^	0.95	2.69	3.6316 (15)	170
C8—H8⋯Br2^i^	0.95	2.68	3.5635 (15)	156

Symmetry code: (i) 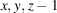 .

**Table 2:** Experimental details

Crystal data	
Chemical formula	C_7_H_14_N_3_^+^·Br^−^
*M*_r_	220.12
Crystal system, space group	Monoclinic, *P*2_1_/*m*
Temperature (K)	100
*a*, *b*, *c* (Å)	8.1283 (2), 21.3822 (7), 8.6376 (2)
β (°)	101.713 (3)
*V* (Å^3^)	1469.96 (7)
*Z*	6
Radiation type	Mo *K*α
μ (mm^−1^)	4.14
Crystal size (mm)	0.38 × 0.25 × 0.04

Data collection	
Diffractometer	Rigaku XtaLAB Synergy-S
Absorption correction	Multi-scan (*CrysAlis PRO*; Rigaku OD; 2022 ▸)
*T*_min_, *T*_max_	0.483, 1.000
No. of measured, independent and observed [*I* > 2σ(*I*)] reflections	23168, 3747, 3142
*R*_int_	0.036
(sin θ/λ)_max_ (Å^−1^)	0.667

Refinement	
*R*[*F* ^2^ > 2σ(*F* ^2^)], *wR*(*F* ^2^), *S*	0.022, 0.052, 1.04
No. of reflections	3747
No. of parameters	168
H-atom treatment	H-atom parameters constrained
Δρ_max_, Δρ_min_ (e Å^−3^)	0.38, −0.29

Computer programs: *CrysAlis PRO* (Rigaku OD, 2022 ▸), *SHELXT* (Sheldrick, 2015*a* ▸), *SHELXL2018/3* (Sheldrick, 2015*b* ▸), *OLEX2* (Dolomanov *et al.*, 2009 ▸), and *publCIF* (Westrip, 2010 ▸).

## full crystallographic data

### Crystal data

**Table:**

C_7_H_14_N_3_^+^·Br^−^	*F*(000) = 672
*M**_r_* = 220.12	*D*_x_ = 1.492 Mg m^−^^3^
Monoclinic, *P*2_1_/*m*	Mo *K*α radiation, λ = 0.71073 Å
*a* = 8.1283 (2) Å	Cell parameters from 9609 reflections
*b* = 21.3822 (7) Å	θ = 3.1–28.2°
*c* = 8.6376 (2) Å	µ = 4.14 mm^−^^1^
β = 101.713 (3)°	*T* = 100 K
*V* = 1469.96 (7) Å^3^	Plate, colourless
*Z* = 6	0.38 × 0.25 × 0.04 mm

### Data collection

**Table:**

Rigaku XtaLAB Synergy-S diffractometer	3747 independent reflections
Radiation source: micro-focus sealed X-ray tube, PhotonJet (Mo) X-ray Source	3142 reflections with *I* > 2σ(*I*)
Mirror monochromator	*R*_int_ = 0.036
Detector resolution: 10.0000 pixels mm^-1^	θ_max_ = 28.3°, θ_min_ = 2.6°
ω scans	*h* = −10→10
Absorption correction: multi-scan (CrysAlis PRO; Rigaku OD; 2022)	*k* = −27→28
*T*_min_ = 0.483, *T*_max_ = 1.000	*l* = −11→11
23168 measured reflections	

### Refinement

**Table:**

Refinement on *F*^2^	Primary atom site location: dual
Least-squares matrix: full	Hydrogen site location: inferred from neighbouring sites
*R*[*F*^2^ > 2σ(*F*^2^)] = 0.022	H-atom parameters constrained
*wR*(*F*^2^) = 0.052	*w* = 1/[σ^2^(*F*_o_^2^) + (0.0235*P*)^2^ + 0.254*P*] where *P* = (*F*_o_^2^ + 2*F*_c_^2^)/3
*S* = 1.04	(Δ/σ)_max_ = 0.001
3747 reflections	Δρ_max_ = 0.38 e Å^−^^3^
168 parameters	Δρ_min_ = −0.29 e Å^−^^3^
0 restraints	

### Fractional atomic coordinates and isotropic or equivalent isotropic displacement parameters (Å^2^)

**Table:**

	*x*	*y*	*z*	*U*_iso_*/*U*_eq_	Occ. (<1)
Br1	0.65535 (3)	0.750000	0.86475 (2)	0.01997 (6)	
Br2	0.36575 (2)	0.58378 (2)	1.16793 (2)	0.01912 (5)	
N1	0.5536 (2)	0.750000	0.34816 (19)	0.0180 (4)	
N2	0.3012 (2)	0.750000	0.22004 (19)	0.0181 (4)	
N3	0.2904 (2)	0.750000	0.3756 (2)	0.0225 (4)	
C1	0.4576 (3)	0.750000	0.2031 (2)	0.0175 (4)	
H1	0.495511	0.750000	0.106069	0.021*	
C2	0.4471 (3)	0.750000	0.4505 (2)	0.0209 (4)	
H2	0.482097	0.750000	0.562368	0.025*	
C3	0.7407 (3)	0.750000	0.3888 (3)	0.0277 (5)	
H3	0.777584	0.750000	0.506529	0.033*	
C4	0.8056 (2)	0.80896 (8)	0.3239 (2)	0.0324 (4)	
H4A	0.772489	0.809040	0.208337	0.049*	
H4B	0.928441	0.810248	0.355041	0.049*	
H4C	0.757925	0.845690	0.366538	0.049*	
C5	0.1468 (3)	0.750000	0.0987 (3)	0.0315 (6)	
H5A	0.079611	0.787411	0.112921	0.038*	0.5
H5B	0.079611	0.712589	0.112921	0.038*	0.5
C6	0.1791 (3)	0.750000	−0.0638 (3)	0.0353 (6)	
H6A	0.225946	0.790528	−0.085598	0.053*	0.5
H6B	0.073605	0.742719	−0.139517	0.053*	0.5
H6C	0.259308	0.716754	−0.073790	0.053*	0.5
N4	0.45707 (16)	0.58697 (5)	0.70657 (14)	0.0163 (3)	
N5	0.71989 (15)	0.59178 (5)	0.80363 (14)	0.0170 (3)	
N6	0.70540 (17)	0.59818 (6)	0.64405 (15)	0.0234 (3)	
C7	0.5439 (2)	0.59462 (7)	0.58810 (18)	0.0223 (3)	
H7	0.493358	0.597046	0.478994	0.027*	
C8	0.57173 (19)	0.58536 (6)	0.84100 (17)	0.0167 (3)	
H8	0.550409	0.580476	0.944460	0.020*	
C9	0.27440 (18)	0.57739 (7)	0.69275 (18)	0.0198 (3)	
H9	0.249096	0.576878	0.801314	0.024*	
C10	0.1780 (2)	0.63095 (8)	0.6001 (2)	0.0296 (4)	
H10A	0.218468	0.670794	0.649669	0.044*	
H10B	0.057966	0.626396	0.599636	0.044*	
H10C	0.195525	0.630178	0.491108	0.044*	
C11	0.2264 (2)	0.51438 (8)	0.61529 (19)	0.0287 (4)	
H11A	0.240093	0.515644	0.505186	0.043*	
H11B	0.109054	0.505046	0.618208	0.043*	
H11C	0.299190	0.481809	0.672508	0.043*	
C12	0.88457 (19)	0.59542 (8)	0.9095 (2)	0.0242 (3)	
H12A	0.917169	0.639848	0.927513	0.029*	
H12B	0.969276	0.574799	0.859048	0.029*	
C13	0.8832 (2)	0.56445 (8)	1.06588 (18)	0.0246 (3)	
H13A	0.847326	0.520823	1.048013	0.037*	
H13B	0.804907	0.586585	1.119266	0.037*	
H13C	0.996325	0.565779	1.132088	0.037*	

### Atomic displacement parameters (Å^2^)

**Table:**

	*U*^11^	*U*^22^	*U*^33^	*U*^12^	*U*^13^	*U*^23^
Br1	0.02633 (12)	0.01928 (12)	0.01483 (11)	0.000	0.00543 (8)	0.000
Br2	0.01946 (8)	0.02267 (9)	0.01542 (8)	−0.00045 (5)	0.00400 (6)	−0.00014 (5)
N1	0.0180 (9)	0.0217 (9)	0.0145 (8)	0.000	0.0036 (7)	0.000
N2	0.0193 (9)	0.0205 (9)	0.0149 (8)	0.000	0.0043 (7)	0.000
N3	0.0242 (10)	0.0261 (10)	0.0194 (9)	0.000	0.0094 (7)	0.000
C1	0.0181 (10)	0.0194 (11)	0.0142 (10)	0.000	0.0015 (8)	0.000
C2	0.0265 (11)	0.0219 (11)	0.0154 (10)	0.000	0.0066 (9)	0.000
C3	0.0196 (11)	0.0469 (15)	0.0148 (10)	0.000	−0.0009 (9)	0.000
C4	0.0238 (8)	0.0396 (10)	0.0359 (9)	−0.0128 (7)	0.0108 (7)	−0.0165 (8)
C5	0.0159 (11)	0.0541 (16)	0.0222 (12)	0.000	−0.0021 (9)	0.000
C6	0.0224 (12)	0.0617 (18)	0.0196 (11)	0.000	−0.0007 (9)	0.000
N4	0.0165 (6)	0.0197 (7)	0.0132 (6)	0.0001 (5)	0.0040 (5)	0.0014 (4)
N5	0.0169 (6)	0.0181 (7)	0.0171 (6)	−0.0005 (5)	0.0059 (5)	0.0009 (4)
N6	0.0244 (7)	0.0275 (7)	0.0204 (7)	−0.0008 (5)	0.0093 (5)	−0.0004 (5)
C7	0.0236 (8)	0.0278 (9)	0.0167 (7)	−0.0001 (6)	0.0068 (6)	−0.0005 (6)
C8	0.0170 (7)	0.0164 (7)	0.0170 (7)	0.0000 (5)	0.0041 (6)	0.0013 (5)
C9	0.0138 (7)	0.0284 (9)	0.0169 (7)	−0.0003 (6)	0.0023 (6)	0.0025 (6)
C10	0.0242 (8)	0.0386 (10)	0.0273 (8)	0.0118 (7)	0.0077 (7)	0.0102 (7)
C11	0.0231 (8)	0.0329 (9)	0.0280 (8)	−0.0061 (7)	0.0003 (7)	−0.0008 (7)
C12	0.0134 (7)	0.0286 (9)	0.0302 (9)	−0.0012 (6)	0.0035 (6)	0.0032 (7)
C13	0.0187 (8)	0.0305 (9)	0.0232 (8)	0.0006 (6)	0.0013 (6)	−0.0004 (6)

### Geometric parameters (Å, º)

**Table:**

N1—C1	1.335 (2)	N4—C7	1.366 (2)
N1—C2	1.357 (3)	N4—C8	1.3337 (19)
N1—C3	1.490 (3)	N4—C9	1.4795 (19)
N2—N3	1.364 (2)	N5—N6	1.3662 (18)
N2—C1	1.308 (3)	N5—C8	1.316 (2)
N2—C5	1.463 (3)	N5—C12	1.4618 (19)
N3—C2	1.307 (3)	N6—C7	1.305 (2)
C3—C4	1.517 (2)	C9—C10	1.520 (2)
C3—C4^i^	1.517 (2)	C9—C11	1.520 (2)
C5—C6	1.480 (3)	C12—C13	1.507 (2)
			
C1—N1—C2	106.43 (18)	C7—N4—C9	128.25 (12)
C1—N1—C3	126.56 (18)	C8—N4—C7	106.21 (13)
C2—N1—C3	127.01 (17)	C8—N4—C9	125.39 (13)
N3—N2—C5	119.20 (18)	N6—N5—C12	120.41 (13)
C1—N2—N3	111.63 (16)	C8—N5—N6	111.24 (12)
C1—N2—C5	129.18 (18)	C8—N5—C12	128.29 (13)
C2—N3—N2	103.67 (18)	C7—N6—N5	104.00 (13)
N2—C1—N1	106.93 (18)	N6—C7—N4	111.31 (13)
N3—C2—N1	111.35 (18)	N5—C8—N4	107.24 (13)
N1—C3—C4^i^	109.14 (11)	N4—C9—C10	109.85 (12)
N1—C3—C4	109.14 (11)	N4—C9—C11	108.78 (12)
C4—C3—C4^i^	112.4 (2)	C11—C9—C10	112.16 (13)
N2—C5—C6	112.79 (19)	N5—C12—C13	111.39 (13)
			
N2—N3—C2—N1	0.000 (1)	N5—N6—C7—N4	0.69 (16)
N3—N2—C1—N1	0.000 (1)	N6—N5—C8—N4	0.42 (15)
N3—N2—C5—C6	180.000 (1)	N6—N5—C12—C13	−158.90 (13)
C1—N1—C2—N3	0.000 (1)	C7—N4—C8—N5	0.01 (15)
C1—N1—C3—C4^i^	−61.59 (13)	C7—N4—C9—C10	−56.70 (19)
C1—N1—C3—C4	61.59 (13)	C7—N4—C9—C11	66.41 (18)
C1—N2—N3—C2	0.000 (1)	C8—N4—C7—N6	−0.46 (16)
C1—N2—C5—C6	0.000 (1)	C8—N4—C9—C10	128.34 (14)
C2—N1—C1—N2	0.000 (1)	C8—N4—C9—C11	−108.55 (15)
C2—N1—C3—C4^i^	118.41 (13)	C8—N5—N6—C7	−0.69 (16)
C2—N1—C3—C4	−118.41 (13)	C8—N5—C12—C13	24.4 (2)
C3—N1—C1—N2	180.000 (1)	C9—N4—C7—N6	−176.18 (13)
C3—N1—C2—N3	180.000 (1)	C9—N4—C8—N5	175.89 (12)
C5—N2—N3—C2	180.000 (1)	C12—N5—N6—C7	−177.94 (13)
C5—N2—C1—N1	180.000 (1)	C12—N5—C8—N4	177.41 (13)

Symmetry code: (i) *x*, −*y*+3/2, *z*.

### Hydrogen-bond geometry (Å, º)

**Table:**

*D*—H···*A*	*D*—H	H···*A*	*D*···*A*	*D*—H···*A*
C1—H1···Br1^ii^	0.95	2.67	3.610 (2)	170
C2—H2···Br2^ii^	0.95	2.70	3.6344 (18)	166
C7—H7···Br2^ii^	0.95	2.69	3.6316 (15)	170
C8—H8···Br2^ii^	0.95	2.68	3.5635 (15)	156

Symmetry code: (ii) *x*, *y*, *z*−1.
